# Effects of canagliflozin on weight loss in high-fat diet-induced obese mice

**DOI:** 10.1371/journal.pone.0179960

**Published:** 2017-06-30

**Authors:** Wenjun Ji, Mei Zhao, Meng Wang, Wenhui Yan, Yuan Liu, Shuting Ren, Jun Lu, Bing Wang, Lina Chen

**Affiliations:** 1 Department of Pharmacology, School of Basic Medical Sciences, Xi’an Jiaotong University Health Science Center, Xi’an, Shaanxi, China; 2 Department of Pharmacy, Taizhou People’s Hospital, Taizhou, Jiangsu, China; 3 Department of Pharmacy, 302 Military Hospital of China, Beijing, China; 4 Department of Pharmacy, Shijiazhuang Maternity Hospital, Shijiazhuang, Hebei, China; 5 Key Laboratory of Environment and Genes Related to Diseases (Xi’an Jiaotong University), Ministry of Education, Xi’an, Shaanxi, China; 6 Department of Pathology, School of Basic Medical Sciences, Xi’an Jiaotong University Health Science Center, Xi’an, Shaanxi, China; 7 Clinical Research Center, the First Affiliated Hospital, Xi’an Jiaotong University Health Science Center, Xi’an, Shaanxi, China; East Tennessee State University, UNITED STATES

## Abstract

Canagliflozin, an inhibitor of sodium glucose co-transporter (SGLT) 2, has been shown to reduce body weight during the treatment of type 2 diabetes mellitus (T2DM). In this study, we sought to determine the role of canagliflozin in body weight loss and liver injury in obesity. C57BL/6J mice were fed a high-fat diet to simulate diet-induced obesity (DIO). Canagliflozin (15 and 60 mg/kg) was administered to DIO mice for 4 weeks. Orlistat (10 mg/kg) was used as a positive control. The body weight, liver weight, liver morphology, total cholesterol (TC) and triglyceride (TG) levels were examined. Signaling molecules, including diacylgycero1 acyltransferase-2 (DGAT2), peroxisome proliferation receptor alpha-1 (PPARα1), PPARγ1, PPARγ2 mRNA levels and the protein expression of SGLT2 were evaluated. Canagliflozin reduced body weight, especially the high-dose canagliflozin, and resulted in increased body weight loss compared with orlistat. Moreover, canagliflozin reduced the liver weight and the ratio of liver weight to body weight, lowered the serum levels of TC and TG, and ameliorated liver steatosis. During the canagliflozin treatment, SGLT2, DGAT2, PPARγ1 and PPARγ2 were inhibited, and PPARα1 was elevated in the liver tissues. This finding may explain why body weight was reduced and secondary liver injury was ameliorated in response to canagliflozin. Together, the results suggest that canagliflozin may be a potential anti-obesity strategy.

## Introduction

Obesity/overweight is a risk factor for cardiovascular disease and type 2 diabetes mellitus (T2DM), among other diseases [1,2]. In 2014, the World Health Organization (WHO) reported that approximately 13% of adults are obese, and 39% of adults are overweight [3]. Additionally, the current anti-obesity drugs often have severe adverse effects. For example, rimonabant increases the incidence of psychiatric side effects, and sibutramine increases blood pressure and the average pulse rate [4,5]. Orlistat, the only available diet pill, has gastrointestinal side effects [6,7]. Therefore, anti-obesity drugs with few side effects are needed.

Most patients with T2DM are obese/overweight. Thus, obesity and T2DM may have a mutual treatment. Canagliflozin, the first sodium glucose co-transporter (SGLT) 2 inhibitor, has been approved by the US FDA to treat T2DM. Interestingly, a recent study has revealed weight loss after administration of canagliflozin in the early treatment of diabetes [8]. Diacylgycerol acyltransferase-2 (DGAT2), peroxisome proliferation receptor alpha-1 (PPARα1), PPARγ1 and PPARγ2 regulate lipid content [9–11]. Therefore, these molecules may be associated with in obesity and liver injury [9,12].

On the basis of previous studies, we hypothesized that canagliflozin might cause weight loss in obese mice. Mice with diet-induced obesity (DIO) were used to assess the changes in body weight and liver injury with canagliflozin, and the underlying mechanisms of weight loss were also examined.

## Materials and methods

### Animals

Four-week-old male C57BL/6J mice (16~19g) were purchased from the Experimental Animal Center of Xi'an Jiaotong University Health Science Center. The animals were housed in plastic cages and given free access to water and food; they were maintained at 22±2°C under automatic 12 h light/12 h dark cycles with 50±5% humidity in a specific-pathogen-free (SPF) environment. The study was approved by the Ethical Committee of Xi’an Jiaotong University and was conducted in accordance with the Practice Guidelines for Laboratory Animals of China.

### Experimental protocols

Canagliflozin was synthesized by Zhejiang Jiaxing Jiuyao Chemical Corporation, and 0.5% carboxymethylcellulose sodium (CMC-Na) was used as the solvent to increase the solubility of canagliflozin.

Mice were randomly divided into a control group (n = 8, fed an ordinary diet) and a HFD group (n = 50, fed a high-fat diet containing 6% fat, 8% water, 18% protein, and 5% fiber) (Huafukang Bioscience Company, Beijing, China). After 4 weeks of feeding, the mice in the HFD group with obesity (the body weight is high over 20% of the mean value of the control mice’ body weight) were considered to be successful DIO mice. DIO mice (n = 32) were intragastrically treated as follows: ① model group: equivalent volume of 0.5% CMC-Na; ② orlistat group (a positive control): orlistat (10 mg/kg/d) (obtained from ZhiEn Pharmaceutic Corporation, Chongqing, China); ③ low-dose canagliflozin (L) group: canagliflozin (15 mg/kg/d); ④ high-dose canagliflozin (H) group: canagliflozin (60 mg/kg/d). The control group was administered the same volume of 0.5% CMC-Na. During the 4-week treatment, the body weight of all mice was measured each day. At the end of the experiment, blood samples were collected from the retro-orbital plexus, and all mice were euthanized by inhalation of overdose diethyl ether in a fume hood, and then cervical dissociation. Sera were separated by centrifugation at 3000 rpm for 15 min and then stored at −80°C. The liver and kidneys were removed and stored at −80°C. After all the procedures were done, the mice carcasses were left in the fume hood for several hours (let ether volatilize), then were stored in a refrigerator and waited to be incinerated.

### Serum levels of TC and TG

Serum levels of total cholesterol (TC) and triglyceride (TG) were measured using mouse TC/TG kits (COD-PAP method, Serial No: A111-2; GPO-PAP method, Serial No: A110-2; Jiancheng Institute of Biotechnology, Nanjing, China), per the manufacturer's instructions.

### Histological analysis

Liver tissues were fixed with 10% neutral-buffered formalin and embedded in paraffin. Then, the samples were cut into 5 μm sections, mounted with hematoxylin–eosin staining and imaged with light microscopy.

### RNA isolation and real time PCR

Total RNA was isolated from mouse livers with TriPure reagents (Roche, Basel, Switzerland). Then, 2 μg RNA was reverse transcribed into cDNA using a Prime Script™ RT Master Mix (Perfect Real Time) (TaKaRa Bio, Inc., Tokyo, Japan). Quantitative real time PCR was performed using SYBR® Premix Ex Taq™ II (TaKaRa Bio, Inc., Tokyo, Japan) with an iQ5 Real-Time PCR Detection System (Bio-Rad Laboratories, Hercules CA). All the primers and probes for real time PCR were purchased from TaKaRa Bio. The specific primers are shown in Table 1. GAPDH served as an endogenous control. The efficiencies of real time PCR for the target genes and the endogenous control were approximately equal.

**Table 1:** Primer sequence used for real-time PCR.

Gene	Forward primer	Reverse primer
DGAT2	5ˈ- ACT TCA CCT GGC TGG CAT TTG -3ˈ	5ˈ- GGT CAG CAG GTT GTG TGT CTT CA -3ˈ
PPARα1	5ˈ- AGT GCC TGT CTG TCG GGA TG -3ˈ	5ˈ- CTC TTG CCC AGA GAT TTG AGG TC -3ˈ
PPARγ1	5ˈ- GGA GCC TAA GTT TGA GTT TGC TGT G -3ˈ	5ˈ- TGC AGC AGG TTG TCT TGG ATG -3ˈ
PPARγ2	5ˈ- GGA GCC TAA GTT TGA GTT TGC TGT G -3ˈ	5ˈ- TGC AGC AGG TTG TCT TGG ATG-3ˈ
GAPDH	5ˈ- TGT GTC CGT CGT GGA TCT GA -3ˈ	5ˈ- TTG CTG TTG AAG TCG CAG GAG -3ˈ

### Western blotting

SGLT2 expression was determined by western blot analysis. Briefly, 100 mg kidney tissue was homogenized in 1 ml RIPA buffer at 12,000×g for 15 min at 4℃. The protein concentration was tested using a bicinchoninic acid (BCA) protein assay kit (Bio-Rad Laboratories, Hercules, CA, USA). Then, 50 μg of protein was separated using 10% SDS/PAGE and transferred to PVDF membranes (Millipore, MA, USA). Subsequently, the membranes were blocked in 5% bovine serum albumin and incubated with primary antibodies against SGLT2 (ab37296, Abcam, Cambridge, UK) and β-actin (sc-47778, Santa Cruz Biotechnology, Inc., Santa Cruz, CA). After incubation with secondary antibody, the bands were analyzed with ChemiDoc XRS. β-actin was used to normalize the results of each sample.

### Statistical analysis

Data are presented as the mean ± standard error of the means (SEM). Differences among groups were determined by ANOVA followed by Tukey’s multiple comparison test with SPSS 16.0 software (SPSS Inc., Chicago, IL, USA). Normal distribution analysis before ANOVA analysis was made with SPSS 16.0 for windows (SPSS Inc, Chicago). Skewness<1 and Kurtosis<1 were considered as normal distribution. Figures were generated with Prism 5.0 software (GraphPad Software Inc., La Jolla, CA, USA). P<0.05 was considered to be significant.

## Results

### Canagliflozin decreased body weight in DIO mice

**Fig 1:**
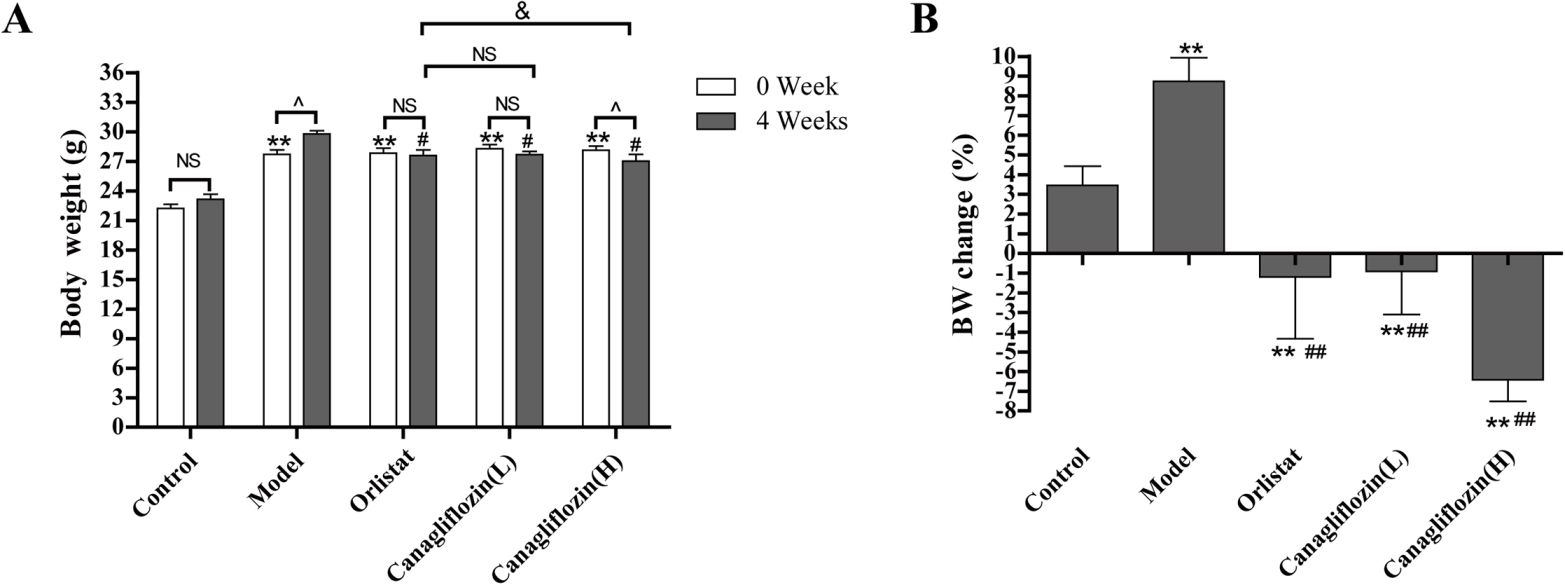
Canagliflozin decreased body weight in DIO mice. (A) Body weight (BW). (B) Body weight change. Data are expressed as the mean ± SEM (n = 8). ** *P* <0.01 vs. control group. # *P* <0.05, ## *P* <0.01 vs. model group. & *P* <0.05 vs. orlistat group. ^ *P* <0.05 vs. pre-treatment group. NS: no statistical significance. Model: diet-induced obesity (DIO); Orlistat: orlistat (10 mg/kg/d); Canagliflozin (L): canagliflozin (15 mg/kg/d); Canagliflozin (H): canagliflozin (60 mg/kg/d).

Initially, DIO mice in the model group were overweight compared with those in the control group. After 4 weeks of treatment, mice in the canagliflozin (H) group showed a reduction in body weight (Fig 1A), the orlistat and canagliflozin (L) groups showed few changes, and the model group showed an increase in body weight. Notably, the canagliflozin (H) group had a lower body weight than that of the orlistat group. Similarly, orlistat- and canagliflozin-treated groups showed body weight loss, in contrast to the model group (Fig 1B). These results indicate that canagliflozin is efficacious in promoting weight loss.

### Canagliflozin inhibited SGLT2 in the kidneys of DIO mice

**Fig 2:**
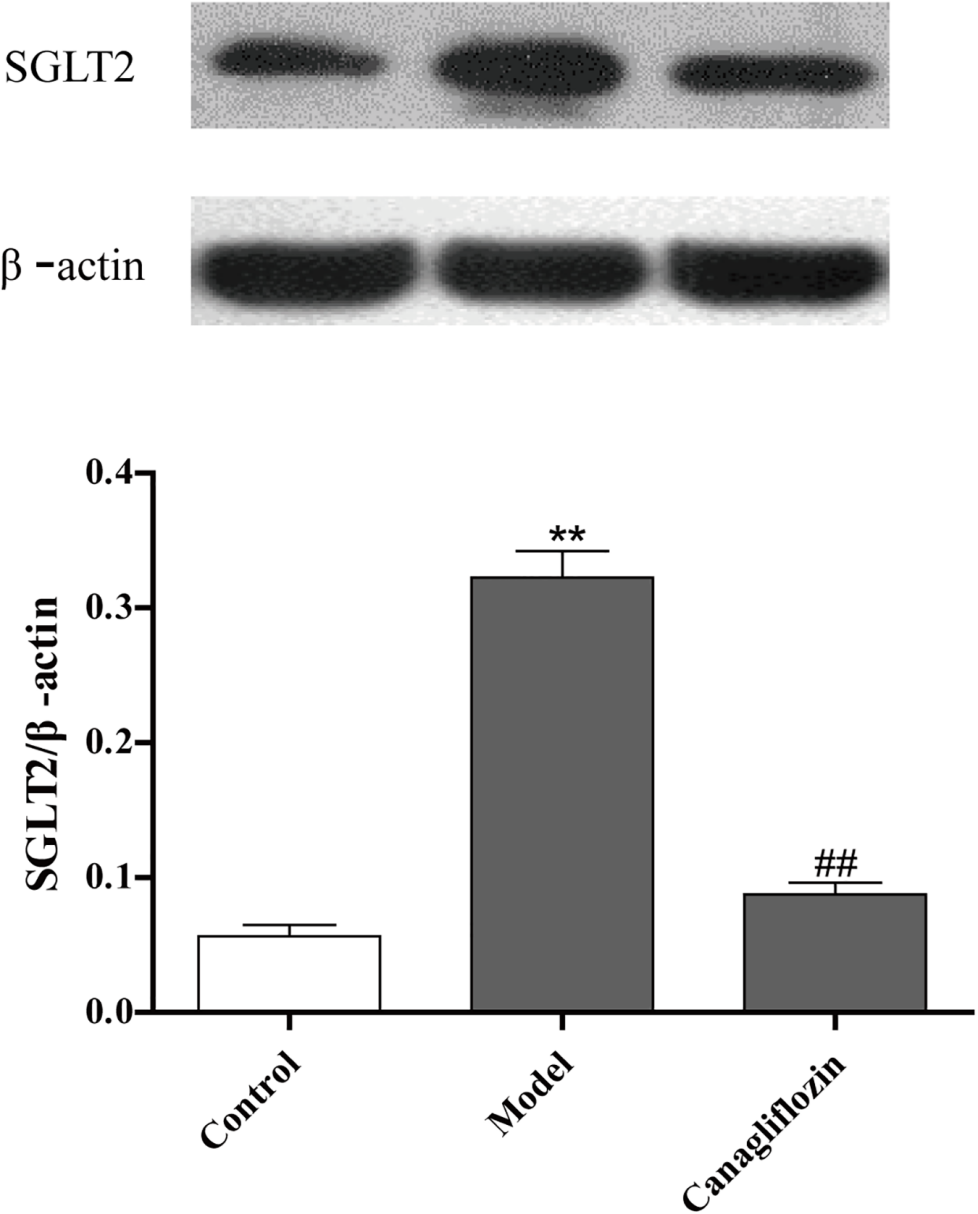
Canagliflozin inhibited SGLT2 expression in the kidneys of DIO mice. Data are expressed as the mean ± SEM (n = 3). ** *P* <0.01 vs. control group. ## *P* <0.01 vs. model group. Model: diet-induced obesity (DIO); Canagliflozin: canagliflozin (15 mg/kg/d).

Because SGLT2 localizes to the early proximal tubule in the kidney, the effect of canagliflozin on SGLT2 was determined in the kidney. DIO mice in the model group, compared with normal mice in the control group, showed a significant increase in SGLT2 expression, whereas the canagliflozin (L) group (/kg/d) exhibited decreased SGLT2 (Fig 2). These data suggested that canagliflozin is a SGLT2 inhibitor in the kidneys of DIO mice.

### Canagliflozin ameliorated liver damage in DIO mice

**Fig 3:**
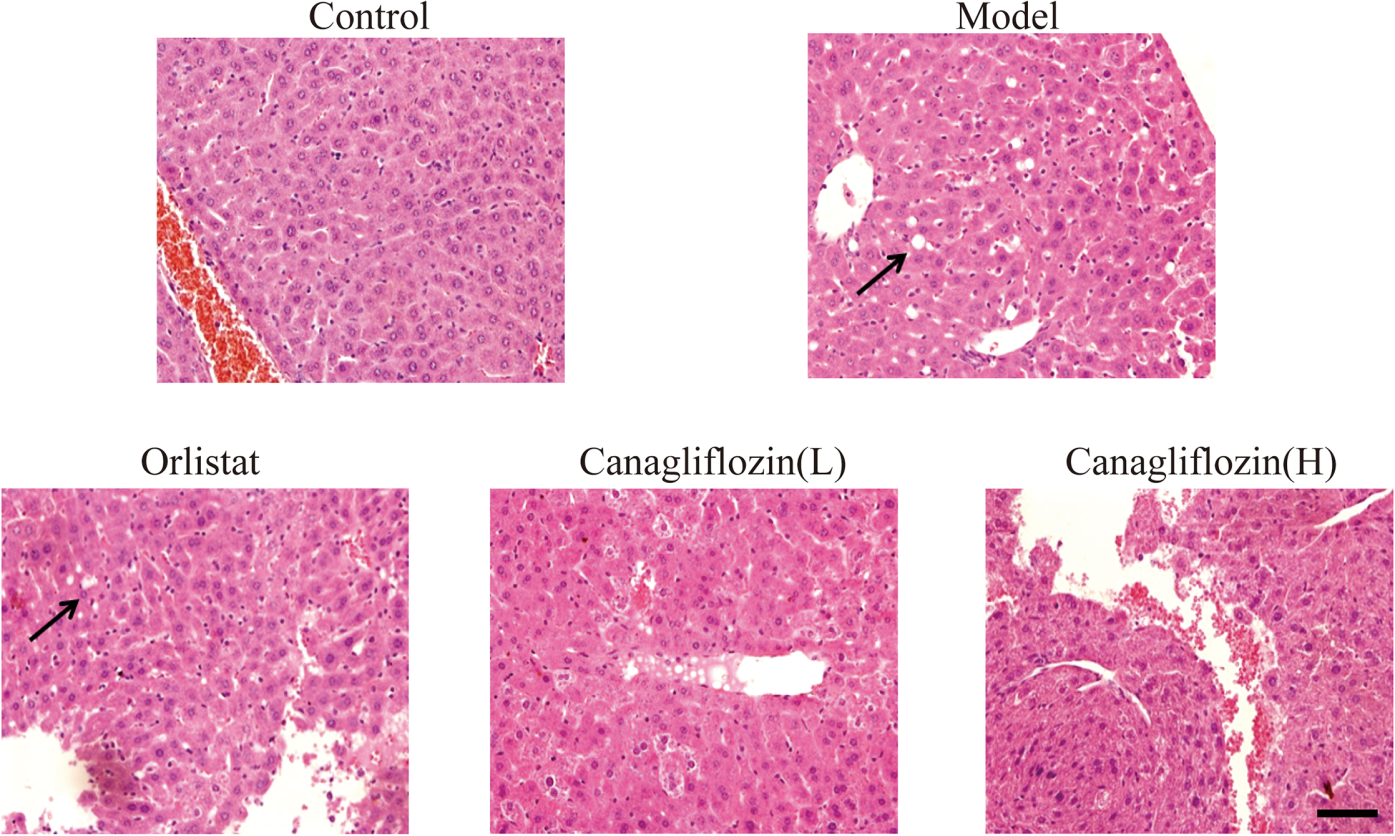
Canagliflozin improved liver morphology in DIO mice. At the end of the experiment, liver tissues were stained with hematoxylin–eosin and observed through light microscopy. Representative images of liver tissue sections are shown (images taken using the 40× objective). Hepatocyte atrophy is indicated by black arrows. Scale bar: 200 μm. Model: diet-induced obesity (DIO); Orlistat: orlistat (10 mg/kg/d); Canagliflozin (L): canagliflozin (15 mg/kg/d); Canagliflozin (H): canagliflozin (60 mg/kg/d).

Obesity is generally accompanied by liver damage. The liver histology of the control mice was normal throughout the 4 weeks of the study. Compared with the control group, the model group exhibited severe hepatocyte atrophy and hepatic steatosis, including cellular and nuclear enlargement, increased lipid droplets and uneven nuclear spacing. The injuries in the DIO mice were ameliorated in drug-treated groups, as shown by an improvement in hepatocyte atrophy and a reduction in the number of lipid droplets, particularly in the canagliflozin groups (Fig 3).

**Fig 4:**
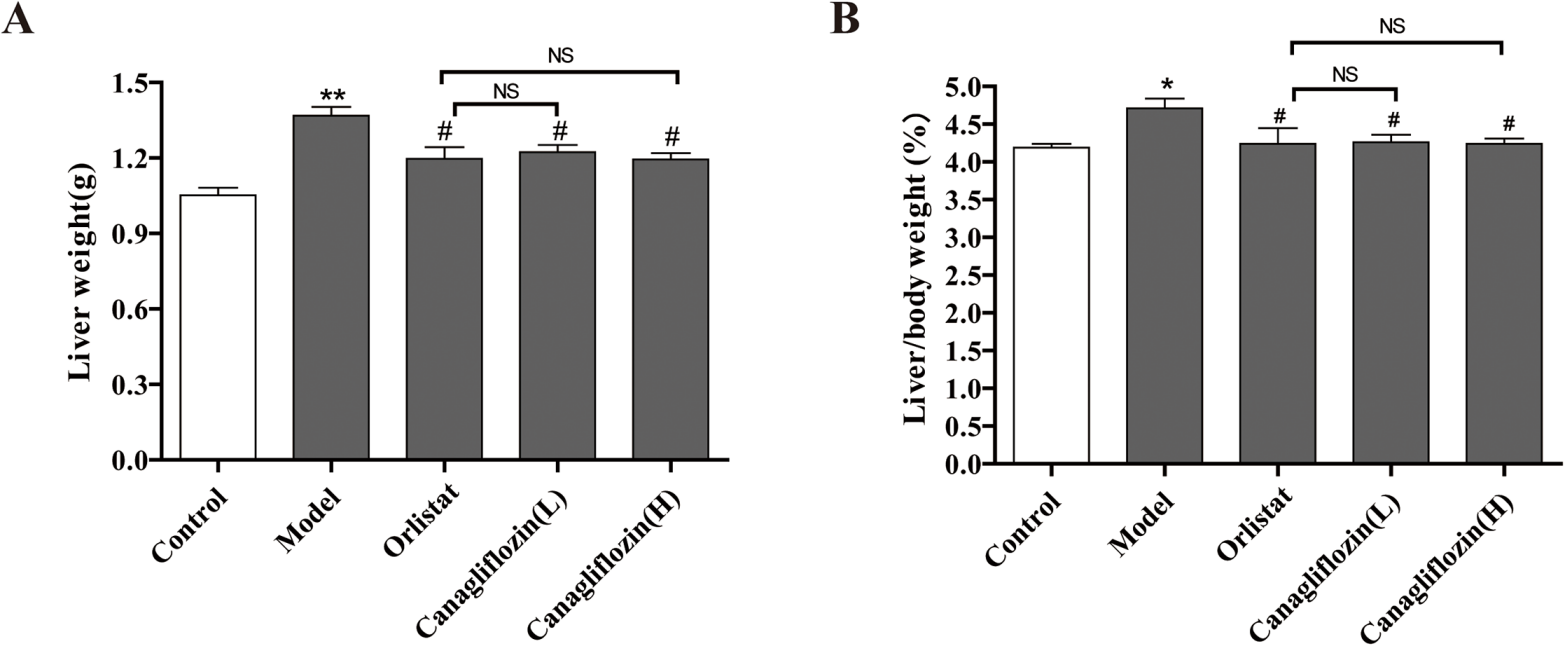
Canagliflozin reduced liver weight and the ratio of liver weight to body weight in DIO mice. (A) Liver weight. (B) The ratio of liver weight to body weight. Data are expressed as the mean ± SEM (n = 8). * *P* <0.05, ** *P* <0.01 vs. control group. # *P* <0.05 vs. model group. NS: no statistical significance. Model: diet-induced obesity (DIO); Orlistat: orlistat (10 mg/kg/d); Canagliflozin (L): canagliflozin (15 mg/kg/d); Canagliflozin (H): canagliflozin (60 mg/kg/d).

The mouse liver weights were further examined. DIO mice in the model group had increased liver weights and increased ratios of liver weight to body weight, by approximately 30% and 12%, respectively compared with the control. After 4 weeks of administration of orlistat and canagliflozin, these effects on liver weight and the ratio of liver weight to body weight were reversed in DIO mice. However, no differences between orlistat and canagliflozin were observed (Fig 4).

### Canagliflozin reduced TC and TG in DIO mice

**Fig 5:**
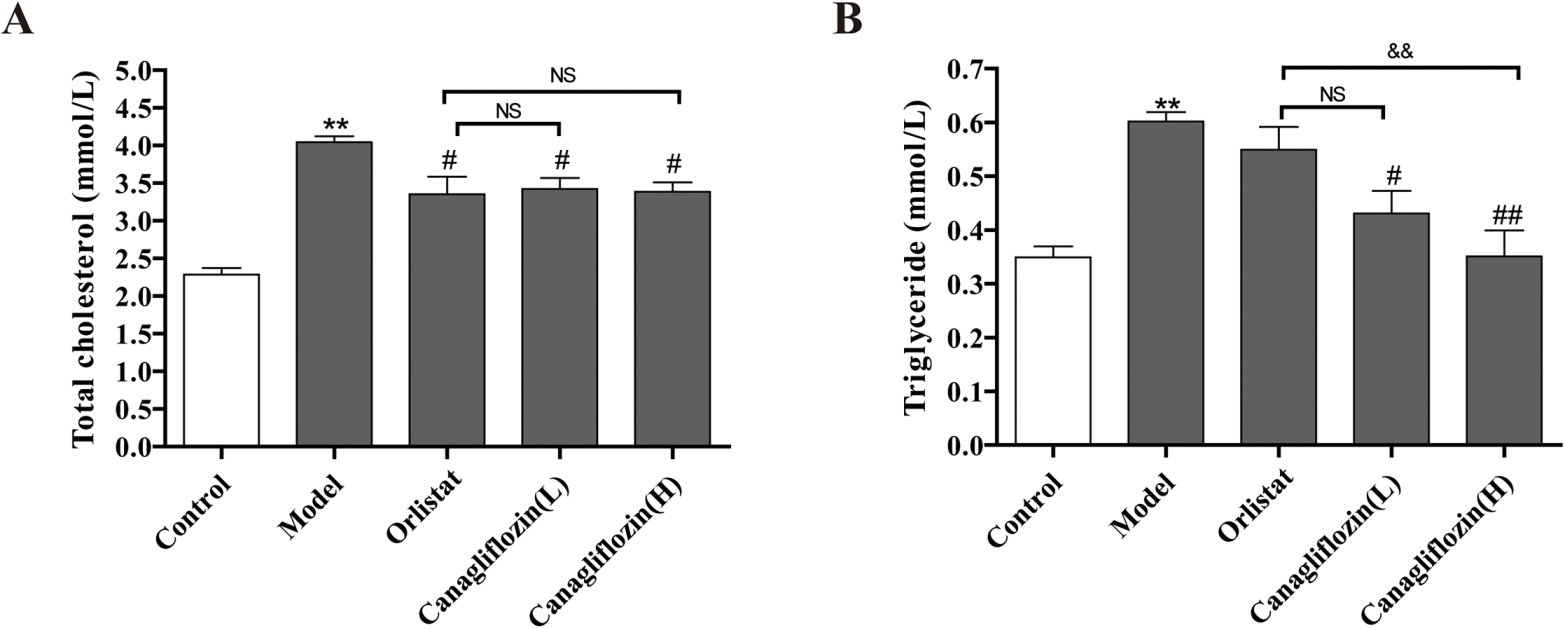
Canagliflozin decreased serum levels of TC and TG in DIO mice. (A) Serum level of total cholesterol (TC). (B) Serum level of triglycerides. Data are expressed as the mean ± SEM (n = 8). ** *P* <0.01 vs. control group. # *P* <0.05, ## *P* <0.01 vs. model group. && *P* <0.01 vs. orlistat group. NS: no statistical significance. Model: diet-induced obesity (DIO); Orlistat: orlistat (10 mg/kg/d); Canagliflozin (L): canagliflozin (15 mg/kg/d); Canagliflozin (H): canagliflozin (60 mg/kg/d).

TC and TG are two important indexes of obesity. As shown in Fig 5, the model group had significantly increased TC and TG levels, thus indicating hyperlipidemia in DIO mice. Orlistat reduced only TC but not TG. Administration of canagliflozin to DIO mice inhibited the TC and TG increases. Moreover, high-dose canagliflozin reversed the increases in TG, which returned to near control levels. These results indicated that canagliflozin-induced weight loss may be associated with decreased serum levels of TC and TG.

### Effects of canagliflozin on DGAT2, PPARα1, PPARγ1 and PPARγ2 expression in DIO mice

**Fig 6:**
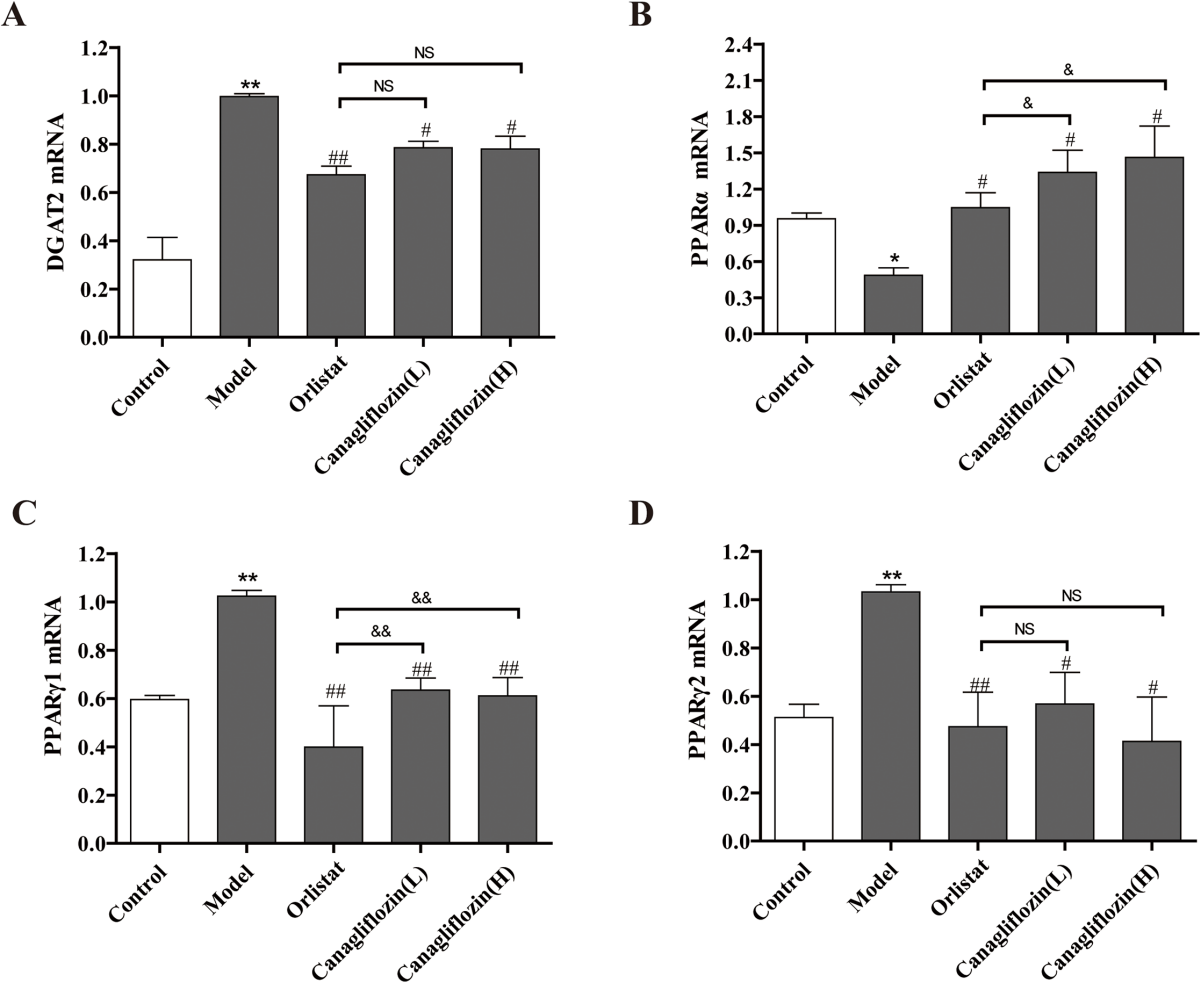
Signaling molecule mRNA expression in DIO mice. (A) DGAT2 mRNA. (B) PPARα mRNA. (C) PPARγ1 mRNA. (D) PPARγ2 mRNA. Data are expressed as the mean ± SEM (n = 3). * *P* <0.05, ** *P* <0.01 vs. control group. # *P* <0.05, ## *P* <0.01 vs. model group. & *P* <0.05, && *P* <0.01 vs. orlistat group. NS: no statistical significance. Model: diet-induced obesity (DIO); Orlistat: orlistat (10 mg/kg/d); Canagliflozin (L): canagliflozin (15 mg/kg/d); Canagliflozin (H): canagliflozin (60 mg/kg/d).

To further confirm the inhibitory effects of canagliflozin on TC and TG, several signaling molecules in the liver tissues were measured by real time PCR. As shown in Fig 6, DGAT2, PPARγ1 and PPARγ2 mRNA were significantly higher in the model group compared with the control group. However, they were reduced when DIO mice were treated with orlistat and canagliflozin. Among these, canagliflozin had a stronger effect on PPARγ1 than orlistat. In contrast, PPARα mRNA in the model group was lower than that in the control, whereas orlistat and canagliflozin treatment reversed this decrease, particularly in the canagliflozin groups.

## Discussion

Canagliflozin has been consistently associated with weight loss in T2DM compared with other anti-T2DM therapies (e.g., biguanides, which are weight-loss neutral, and pioglitazone, which causes weight gain) [8,13,14]. In this study, we demonstrated that canagliflozin decreased body weight in high-fat diet-induced obese C57BL/6J mice. Furthermore, canagliflozin ameliorated liver injury by reducing the liver weight and ratio of liver weight to body weight, lowering the serum levels of TC and TG and ameliorating liver steatosis. During the treatment with canagliflozin, DGAT2, PPARγ1 and PPARγ2 were reduced, and PPARα1 was elevated in the liver tissues. These findings may explain why body weight was reduced and liver injury ameliorated in response to canagliflozin.

Weight reduction is an additional benefit induced by canagliflozin when it is administered along with insulin [15]. Canagliflozin monotherapy also reduces mean body weight in T2DM patients [16]. The current results, although they were obtained in non-diabetic mice, provide direct evidence of weight reduction. Moreover, SGLT2 expression in the kidney was inhibited by canagliflozin, thus suggesting that SGLT2 may participate in the body weight decrease. High-fat diets induces hepatic steatosis and insulin resistance, as occurs during the development of human obesity [17]. In DIO mice, a large number of lipid droplets were observed in the liver. Moreover, the liver weights and ratios of liver weight to body weight in the high-fat group were significantly elevated, results consistent with those of previous studies [18,19]. Notably, canagliflozin administration for 4 weeks mitigated the changes in the liver weight and the ratio of liver weight to body weight. Moreover, canagliflozin prevented severe hepatocyte atrophy and hepatic steatosis in DIO mice. From these results, we propose that the anti-obesity effect of canagliflozin may be mediated by decreasing liver weight and the ratio of liver weight to body weight and by ameliorating liver damage.

TC and TG, which are predominantly synthesized in liver, are important markers of lipid metabolic disorders [20–22]. Ipragliflozin, a selected SGLT2 inhibitor, improves steatosis and decreases TC levels in the liver [23–25], thus suggesting that a SGLT2 inhibitor may limit lipoprotein development. Here, we confirmed that canagliflozin prevented the increased TC and TG in DIO mice. Notably, canagliflozin, compared with orlistat, resulted in a greater decrease in serum TG.

DGAT2 is an integral membrane protein that promotes synthesis of TG and its storage in lipid droplets [10,26,27]. We found that DGAT2 mRNA in the liver tissues of DIO mice was elevated, whereas canagliflozin reversed this effect. The data indicated that canagliflozin suppressed the synthesis of TG and ultimately the accumulation of hepatic lipid droplets via down-regulation of DGAT2. PPARα1 is involved in energy metabolism, and PPARγ is associated with lipogenesis [28]. The higher PPARα1 and lower PPARγ1 and PPARγ2 levels were observed after canagliflozin treatment may help prevent liver damage. The data also indicated that canagliflozin had a stronger effect on PPAR regulation than orlistat.

## Conclusions

Our results demonstrated that canagliflozin therapy for 4 weeks decreased body weight, liver weight and the liver weight/body weight ratio and inhibited the serum accumulation of TC and TG. In addition to inhibition of SGLT2, the improvement in hepatic steatosis and lipid synthesis may be mediated via up-regulation of PPARα1 and down-regulation of DGAT2, PPARγ1 and PPARγ2. Because canagliflozin was shown to induce weight loss and hypoglycemia, it may hold promise as an anti-obesity drug. However, longer durations of canagliflozin treatment on obesity and the underlying mechanism should be studied in the future.

## Supporting information

S1 Raw Data (DOCX)
