# Supplementary material for: Effects of canagliflozin on weight loss in high-fat diet-induced obese mice
Source: PLoS One. 2017 Jun 30;12(6):e0179960. doi: 10.1371/journal.pone.0179960 (PMC5493335; doi:10.1371/journal.pone.0179960)
Supplement: S1 Raw Data — (DOCX) [file pone.0179960.s001.docx]

**Raw data as supplemental information of manuscript (PONE-D-16-36467R2_FTC2)**

**Fig 1. Canagliflozin decreased body weight in DIO mice.**

**Body weight (g)**

Before treatment

|  | 1 | 2 | 3 | 4 | 5 | 6 | 7 | 8 |
| --- | --- | --- | --- | --- | --- | --- | --- | --- |
| Control | 23.5 | 23 | 22.5 | 21.5 | 19.5 | 23.5 | 23 | 22 |
| Model | 29.5 | 29 | 28 | 27 | 29 | 28 | 27 | 26 |
| Orlistat | 30.5 | 29 | 28 | 27 | 29 | 28 | 27 | 26 |
| Canagliflozin (L) | 29 | 29 | 28 | 27 | 29 | 28 | 27 | 26 |
| Canagliflozin (H) | 29 | 28 | 27.5 | 27 | 26.5 | 29 | 28 | 27 |

After treatment for 4 weeks

|  | 1 | 2 | 3 | 4 | 5 | 6 | 7 | 8 |
| --- | --- | --- | --- | --- | --- | --- | --- | --- |
| Control | 24.5 | 23 | 22.5 | 22.5 | 19.5 | 25.5 | 23 | 23 |
| Model | 30 | 30 | 29.5 | 30 | 28.5 | 30 | 29 | 32 |
| Orlistat | 29 | 28.5 | 28.5 | 26.5 | 29 | 26 | 29 | 26 |
| Canagliflozin (L) | 28 | 26 | 27.5 | 27.5 | 27.5 | 26.5 | 28 | 24.5 |
| Canagliflozin (H) | 26 | 31 | 25 | 27 | 24 | 28.5 | 26.5 | 26.5 |

**Fig 2. Canagliflozin inhibited SGLT2 expression in the kidneys of DIO mice.**

|  | mean | SE |
| --- | --- | --- |
| Control | 0.056427 | 0.00842323 |
| Model | 0.3226141 | 0.01954234 |
| Canagliflozin | 0.0876465 | 0.00867702 |

**Fig 4. Canagliflozin reduced liver weight and the ratio of liver weight to body weight in DIO mice.**

**Liver weight (g)**

|  | 1 | 2 | 3 | 4 | 5 | 6 | 7 | 8 |
| --- | --- | --- | --- | --- | --- | --- | --- | --- |
| Control | 0.973 | 1.151 | 1.181 | 0.957 | 1.036 | 1.101 | 0.968 | 1.048 |
| Model | 1.327 | 1.498 | 1.486 | 1.316 | 1.205 | 1.333 | 1.442 | 1.335 |
| Orlistat | 1.283 | 1.283 | 1.261 | 1.271 | 1.083 | 1.162 | 1.262 | 1.087 |
| Canagliflozin (L) | 1.221 | 1.244 | 1.129 | 1.175 | 1.243 | 1.332 | 1.241 | 1.204 |
| Canagliflozin (H) | 1.108 | 1.239 | 1.144 | 1.176 | 1.178 | 1.21 | 1.229 | 1.268 |

**Fig 5. Canagliflozin decreased serum levels of TC and TG in DIO mice.**

**A. TC (mmol/L)**

|  | 1 | 2 | 3 | 4 | 5 | 6 | 7 | 8 |
| --- | --- | --- | --- | --- | --- | --- | --- | --- |
| Control | 1.899132 | 2.647121 | 2.335248 | 2.373281 | 2.053801 | 2.112119 | 2.728259 | 1.998019 |
| Model | 3.874331 | 4.127886 | 4.201417 | 3.813477 | 4.30284 | 3.930113 | 4.144711 | 3.93864 |
| Orlistat | 3.298759 | 3.757695 | 3.696841 | 3.592884 | 2.228754 | 3.526959 | 3.703253 | 2.997382 |
| Canagliflozin (L) | 2.903212 | 3.374826 | 3.732339 | 3.674021 | 3.423002 | 3.412752 | 3.627026 | 3.213021 |
| Canagliflozin (H) | 3.113664 | 3.083237 | 3.293688 | 3.62331 | 3.643595 | 3.045203 | 3.897121 | 3.3857617 |

**B. TG (mmol/L)**

|  | 1 | 2 | 3 | 4 | 5 | 6 | 7 | 8 |
| --- | --- | --- | --- | --- | --- | --- | --- | --- |
| Control | 0.3763427 | 0.3211354 | 0.2842009 | 0.288943 | 0.3926716 | 0.3876541 | 0.365384 | 0.378559 |
| Model | 0.6018373 | 0.5711233 | 0.6318373 | 0.652488 | 0.622617 | 0.562023 | 0.568247 | 0.602596 |
| Orlistat | 0.6212764 | 0.5594598 | 0.5886186 | 0.4268846 | 0.562898 | 0.537668 | 0.583784 | 0.511887 |
| Canagliflozin (L) | 0.4634302 | 0.311027 | 0.4463238 | 0.5030862 | 0.454612 | 0.472471 | 0.392431 | 0.404356 |
| Canagliflozin (H) | 0.2783692 | 0.307528 | 0.5427421 | 0.3180251 | 0.3086963 | 0.288684 | 0.397344 | 0.367184 |

**Fig 6. Signaling molecule mRNA expression in DIO mice.**

|  | DGAT2 | | PPARα | | PPARγ1 | | PPARγ2 | |
| --- | --- | --- | --- | --- | --- | --- | --- | --- |
|  | mean | SE | mean | SE | mean | SE | mean | SE |
| Control | 0.320589467 | 0.131740106 | 0.933310267 | 0.047421922 | 0.595481867 | 0.014162378 | 0.511140267 | 0.049914565 |
| Model | 0.996828333 | 0.016760908 | 0.4849695 | 0.088775421 | 1.000283133 | 0.00185948 | 1.000905567 | 0.000680343 |
| Orlistat | 0.6724162 | 0.050898741 | 1.045268333 | 0.175130399 | 0.398231667 | 0.242394993 | 0.473655567 | 0.202459032 |
| Canagliflozin (L) | 0.784369767 | 0.038251113 | 1.369491667 | 0.156043745 | 0.6349048 | 0.070669329 | 0.567835133 | 0.184959913 |
| Canagliflozin (H) | 0.779783 | 0.074615763 | 1.461938433 | 0.365704472 | 0.610694367 | 0.106545028 | 0.412828567 | 0.260019191 |
